# Supplementary material for: All electromagnetic scattering bodies are matrix-valued oscillators
Source: Nat Commun. 2023 Nov 24;14:7724. doi: 10.1038/s41467-023-43221-2 (PMC10673840; doi:10.1038/s41467-023-43221-2)
Supplement: Supplementary file 1 — Supplementary Information [file 41467_2023_43221_MOESM1_ESM.pdf]

Supplementary Materials:
All electromagnetic scattering bodies are matrix-valued oscillators

Lang Zhang, Francesco Monticone, and Owen D. Miller
(Dated: October 25, 2023)

CONTENTS

Supplementary Note 1. Integral-operator definition of the $\mathbb{T}$ matrix 2

Supplementary Note 2. Complex-frequency symmetries of the $\mathbb{T}$ matrix 2

Supplementary Note 3. Oscillator representation via Herglotz functions 3
A. Background: passive linear systems 3
B. Alternative derivation of the integral representation of the main text 5

Supplementary Note 4. Physical oscillators vs. mathematical oscillators 6

Supplementary Note 5. Low-frequency near-field sum rule for the $\mathbb{T}$ matrix 8

Supplementary Note 6. Scattering simulation parameters for Fig. 1 9

Supplementary Note 7. Optimal frequencies of NFRHT for state-of-the-art materials 9

References 9

Supplementary Note 1. INTEGRAL-OPERATOR DEFINITION OF THE $\mathbb{T}$ MATRIX

In the main text, we used linearity as a sufficient condition to argue that the polarization fields $\mathbf{P}(\mathbf{x})$ induced by an incident field $\mathbf{E}_{\text{inc}}(\mathbf{x})$ must be related through a linear operator that one can call "$\mathbb{T}$:"

$$\mathbf{P}(\mathbf{x}) = \int_V \mathbb{T}(\mathbf{x}, \mathbf{x}')\mathbf{E}_{\text{inc}}(\mathbf{x}')\, \mathrm{d}\mathbf{x}', \tag{1}$$

or, in our vector notation,

$$\mathbf{p} = \mathbb{T}\mathbf{e}_{\text{inc}}. \tag{2}$$

In this section, we discuss the (known [1]) *construction* of the $\mathbb{T}$ matrix from known integral-equation operators.

For any scattering problem, the volume (Lippmann–Schwinger) integral equation is [2]

$$\int_V \mathbb{G}_0(\mathbf{x}, \mathbf{x}')\mathbf{P}(\mathbf{x}')\, \mathrm{d}\mathbf{x}' - \frac{1}{\chi(\mathbf{x})}\mathbf{P}(\mathbf{x}) = -\mathbf{E}_{\text{inc}}(\mathbf{x}), \tag{3}$$

where the first term is the scattered field and the second term is the negative of the total field. Or, in vector notation,

$$\left[\mathbb{G}_0 - \chi^{-1}\right] \mathbf{p} = -\mathbf{e}_{\text{inc}}. \tag{4}$$

The $\mathbb{T}$ matrix, then, is the negative inverse of the matrix in square brackets:

$$\mathbb{T} = -\left[\mathbb{G}_0 - \chi^{-1}\right]^{-1}. \tag{5}$$

Hence the $\mathbb{T}$ matrix can be computed via standard methods [2].

Supplementary Note 2. COMPLEX-FREQUENCY SYMMETRIES OF THE $\mathbb{T}$ MATRIX

For an electric field or a polarization field, the usual symmetry relation is $\mathbf{E}(-\omega^*) = \mathbf{E}^*(\omega)$ and $\mathbf{P}(-\omega^*) = \mathbf{P}^*(\omega)$, which are consequences of the real-valued nature of the time-domain fields (proven by Fourier transform). By exactly the same reasoning for the $\mathbb{T}$ matrix, we have:

$$\mathbb{T}(-\omega^*) = \mathbb{T}^*(\omega), \tag{6}$$

where the matrix asterisk denotes entrywise conjugation. The entrywise nature of Eq. (6) inhibits direct symmetry relations for the Hermitian and anti-Hermitian parts of $\mathbb{T}$. The key, then, is to break the $\mathbb{T}$ matrix into its complex-symmetric and skew-symmetric parts, which we can refer to as its "reciprocal" and "nonreciprocal" (or, really, "anti-reciprocal") parts:

$$\mathbb{T} = \mathbb{R} + \mathbb{N}, \tag{7}$$

where $\mathbb{R} = (\mathbb{T} + \mathbb{T}^T)/2$, and $\mathbb{N} = (\mathbb{T} - \mathbb{T}^T)/2$. Then one immediately has the same symmetry relations for $\mathbb{R}$ and $\mathbb{N}$, i.e., $\mathbb{R}(-\omega^*) = \mathbb{R}^*(\omega)$ and $\mathbb{N}(-\omega^*) = -\mathbb{N}^*(\omega)$, but they can be directly converted to matrix symmetry relations:

$$\begin{aligned} \mathbb{R}(-\omega^*) &= \mathbb{R}^\dagger(\omega), \\ \mathbb{N}(-\omega^*) &= -\mathbb{N}^\dagger(\omega). \end{aligned} \tag{8}$$

From these, one can immediately read off the symmetry relations for the Hermitian and anti-Hermitian parts of $\mathbb{R}$ and $\mathbb{N}$: $\operatorname{Re}\mathbb{R}(-\omega^*) = \operatorname{Re}\mathbb{R}(\omega)$, $\operatorname{Im}\mathbb{R}(-\omega^*) = -\operatorname{Im}\mathbb{R}(\omega)$, $\operatorname{Re}\mathbb{N}(-\omega^*) = -\operatorname{Re}\mathbb{N}(\omega)$, and $\operatorname{Im}\mathbb{N}(-\omega^*) = \operatorname{Im}\mathbb{N}(\omega)$.

Hence neither $\mathbb{R}(\omega)$ nor $\mathbb{N}(\omega)$ have additional degrees of freedom at negative (real) frequencies; their positive-frequency components represent all of their independent degrees of freedom. We can use these symmetries to prove the positive-definiteness conditions on the reciprocal and nonreciprocal parts of the response. In the main text, we defined at real frequencies: $\omega \operatorname{Im}\mathbb{T}(\omega) = \mathbb{X}(\omega) + \mathbb{Y}(\omega)$, where $\mathbb{X}$ and $\mathbb{Y}$ are the reciprocal and nonreciprocal parts of $\omega \operatorname{Im}\mathbb{T}(\omega)$, which means that $\mathbb{X}(\omega) = \omega \operatorname{Im}\mathbb{R}(\omega)$ and $\mathbb{Y}(\omega) = \omega \operatorname{Im}\mathbb{N}(\omega)$. At nonnegative frequencies, the positive semidefinite property of $\omega \operatorname{Im}\mathbb{T}(\omega)$ directly implies that

$$\mathbb{X}(\omega) + \mathbb{Y}(\omega) \geq 0. \tag{9}$$

At negative frequencies ($-\omega$, for ω positive), we similarly have:

$$\mathbb{X}(-\omega) + \mathbb{Y}(-\omega) \geq 0. \tag{10}$$

But we can use the symmetry relations above to simplify this expression to positive frequencies. From the relations for $\mathbb{R}(\omega)$ and $\mathbb{N}(\omega)$, it follows that for real-valued frequencies, $\mathbb{X}(-\omega) = \mathbb{X}(\omega)$ and $\mathbb{Y}(-\omega) = -\mathbb{Y}(\omega)$. Hence the negative-frequency positivity condition can be converted to:

$$\mathbb{X}(\omega) - \mathbb{Y}(\omega) \geq 0. \tag{11}$$

Hence at every nonnegative frequency, $\mathbb{X} + \mathbb{Y}$ and $\mathbb{X} - \mathbb{Y}$ must be positive semidefinite; these two conditions also imply that $\mathbb{X}$ itself must be positive semidefinite.

Supplementary Note 3. OSCILLATOR REPRESENTATION VIA HERGLOTZ FUNCTIONS

In this section we include (a) a survey of representations of passive linear systems, and (b) an alternative derivation of Eq. (3) of the main text, using a Herglotz–Nevanlinna representation.

A. Background: passive linear systems

There is a long history of identifying constraints associated with passive linear systems [3–10], with applications in fields ranging from circuit theory and control [11] to electrical interconnects [12] to elastic materials [13] to quantum field theory [14]. In this section, we review some of the classic results of what is known in passive linear systems. We will specialize to N-port systems, in which there is a finite number N of orthogonal input/output channels that are normalized to each carry unit power into or out of the system. Particularly useful pedagogical introductions include Refs. [6, 8, 10, 12].

We can start with the "scattering" picture of a linear system. Linearity implies that input amplitudes, collated into an $N \times 1$ vector $\mathbf{a}$, are scattered into an $N \times 1$ vector of output amplitudes $\mathbf{b}$ that can be found via an $N \times N$ scattering matrix $\mathbb{S}$:

$$\mathbf{b} = \mathbb{S}\mathbf{a}. \tag{12}$$

Passivity is the condition that that the total outflow of power up to any time t be smaller than the total inflow of power up to the same time:

$$\int_{-\infty}^{t} \left[\mathbf{a}^T(\tau)\mathbf{a}(\tau) - \mathbf{b}^T(\tau)\mathbf{b}(\tau)\right] \, \mathrm{d}\tau \geq 0. \tag{13}$$

This condition is sometimes referred to as *strong passivity*, and its *weak passivity* counterpart is defined *only* for the limit in which $t \to \infty$. The results below do not necessarily hold in weakly passive systems without separately invoking a causal scattering operator [8]. By contrast, strong passivity actually *implies* causality. Causality can be defined as the requirement that a zero input signal, $\mathbf{a}(\tau) = 0$, up to some time t, implies zero output, $\mathbf{b}(\tau) = 0$ up to the same time; this condition is an immediate consequence of Eq. (13).

Whereas the conventional engineering literature where much of passivity theory originated uses the Laplace domain, we will use the frequency domain to conform with typical modern scattering theory. The key consequence is that the right-half plane (RHP) is rotated to the upper-half plane (UHP), and some of the key results will refer to positive imaginary parts (suitably defined) in the UHP, instead of positive real parts in the RHP. We will overload the same variable names for time- and frequency-domain versions of a variable, with the argument denoting the context. For example, the time domain version of Eq. (12) is $\mathbf{b}(t) = \int \mathbb{S}(t-t')\mathbf{a}(t')\,\mathrm{d}t'$, and the frequency-domain version is $\mathbf{b}(\omega) = \mathbb{S}(\omega)\mathbf{a}(\omega)$.

The first key result of passivity declares necessary and sufficient conditions of the scattering matrix $\mathbb{S}$ of a passive linear system. In particular, $\mathbb{S}(\omega)$ is the frequency-domain scattering matrix of a passive linear system if and only if:

1. $\mathbb{S}(\omega)$ is analytic for $\operatorname{Im}\omega > 0$,

2. $\mathbb{I} - \mathbb{S}^\dagger(\omega)\mathbb{S}(\omega)$ is positive semidefinite for $\operatorname{Im}\omega > 0$, and

3. $\mathbb{S}^*(\omega) = \mathbb{S}(-\omega^*)$,

where $\mathbb{I}$ is the $N \times N$ identity matrix. These conditions define *bounded-real* functions [4, 12]. A simple physical interpretation of the conditions is that the first is a consequence of causality, the second a consequence of passivity (for inputs with both oscillating and growth/decay terms), and the third is a consequence of real-valued time-domain signals, though such an interpretation only implies that they are necessary, and not their sufficiency. The proofs in the literature tend to be rigorous but also quite formal. One issue with these conditions is that they require certain properties to be satisfied over the entire half-space of the UHP, which can be computationally expensive for applications such as real-time passivity verification [9]. There is an alternative characterization entirely using real-line values [6, 12]. It is not so important for our work, so we will not highlight it, but the characterization essentially boils down to three conditions: $\mathbb{S}(\omega)$ satisfies Kramers–Kronig relations, $I - \mathbb{S}^\dagger(\omega)\mathbb{S}(\omega)$ is positive semidefinite everywhere on the real line, and $\mathbb{S}(\omega) = \mathbb{S}^*(-\omega)$. Alternatively, and closer to our interests, is a representation theorem for scattering matrices. The scattering matrix of a passive linear system everywhere in the UHP can be written:

$$\mathbb{S}(\omega = \omega_0 + i\gamma) = \frac{\gamma}{\pi}\int_{-\infty}^{\infty} \frac{\mathbb{S}(\omega')}{(\omega' - \omega_0)^2 + \gamma^2}\,\mathrm{d}\omega'. \tag{14}$$

More rigorous derivations of Eq. (14) start with the function $\mathbb{S}(\omega)$ defined only in the UHP, then proves Eq. (14) where $\mathbb{S}(\omega')$ are boundary values of the function, suitably defined [8]. An alternative, slightly less rigorous approach, is to use the analyticity of $\mathbb{S}(\omega)$ and take a contour integral of $\mathbb{S}(\omega)/\left[(\omega - \omega_0)^2 + \gamma^2\right]$.

The conditions above represent the key conclusions of passivity in a *scattering* formalism (mapping inputs to outputs). They can be quite useful for validation [9], i.e., verifying that a scattering matrix represents a passive system, but they are less useful from a theoretical bound perspective. One issue is that it appears difficult to identify a sum rule for a positive-definite quantity from which $\mathbb{S}(\omega)$ can be built. An even more significant issue is a subtle one: the simple definition of Eq. (12) can in fact be difficult to realize: one needs a basis of independent "channels" on which to define $\mathbf{a}(t)$ and $\mathbf{b}(t)$, but typical basis functions (e.g. vector spherical waves) are spatially distributed, which leads to more complex passivity and causality conditions. In particular, any definition of causality requires the introduction of phase shifts related to the properties of the physical scatterer [15]. This appears to render impossible any hope of a scattering-matrix-based framework for spectral bounds.

Of more utility for our purposes is the *immittance* formalism, in which the port variables are currents $\mathbf{i}$ and voltages $\mathbf{v}$. "Immittance" refers to the class of matrices representing either impedances or admittances, which have identical necessary and sufficient passivity conditions in many cases. Abstractly, the immittance variables can be derived from the scattering variables, as $\mathbf{v} = 2\left(\mathbf{a} - \mathbf{b}\right)$ and $\mathbf{i} = 2\left(\mathbf{a} + \mathbf{b}\right)$, or vice versa. The strong passivity condition of Eq. (13) is then, in the immittance variables,

$$\int_{-\infty}^{t} \mathbf{v}^T(\tau)\mathbf{i}(\tau)\,\mathrm{d}\tau \geq 0. \tag{15}$$

An immittance matrix $\mathbb{X}$ (representing an impedance $\mathbb{Z}$ or an admittance $\mathbb{Y}$) represents an N-port linear system if and only if:

1. $\mathbb{X}(\omega)$ is analytic for $\operatorname{Im}\omega > 0$,
2. $\operatorname{Re}\mathbb{X}(\omega) = \frac{1}{2}\left[\mathbb{X}(\omega) + \mathbb{X}^\dagger(\omega)\right]$ is positive semidefinite for $\operatorname{Im}\omega > 0$, and
3. $\mathbb{X}^*(\omega) = \mathbb{X}(-\omega^*)$.

These conditions define *positive-real* matrices. (Sometimes, though not always [4], they are only defined as such in the Laplace domain.) They are identical to the conditions for the scattering matrix, except that the passivity condition is now in the Hermitian part of the immittance matrix, whereas the relevant quantities for scattering matrices is $\mathbb{I} - \mathbb{S}^\dagger(\omega)\mathbb{S}(\omega)$. The analogous real-line-only conditions for the impedance matrix are less insightful than those for scattering matrices [6, 12], so we do not include them here. Alternatively, there is a quite useful representation theorem for immitance matrices, although for compatibility with our $\mathbb{T}$-matrix discussions, we will first make a small pivot. A $\mathbb{T}$ matrix relates a field to a dipole density, rather than a current, and hence a $\mathbb{T}$ matrix is analogous to an immittance matrix multiplied by frequency and the imaginary unit i. Hence, a $\mathbb{T}(\omega)$ matrix represents a passive N-port linear system if and only if:

1. $\omega\mathbb{T}(\omega)$ is analytic for $\operatorname{Im}\omega > 0$,
2. $\operatorname{Im}\left[\omega\mathbb{T}(\omega)\right]$ is positive semidefinite for $\operatorname{Im}\omega > 0$, and
3. $\omega^*\mathbb{T}^*(\omega) = -\left[\omega\mathbb{T}(\omega)\right]_{\omega=-\omega^*}$,

where $\mathrm{Im}\,[\omega\mathbb{T}(\omega)]$ is the anti-Hermitian part of $\omega\mathbb{T}(\omega)$. The first two of these conditions defines a matrix-valued *Herglotz–Nevanlinna* function. There is a well-known representation theorem for such functions [16–18]:

$$\omega\mathbb{T}(\omega) = \mathbb{C} + \mathbb{D}\omega + \int_{-\infty}^{\infty} \left[\frac{1}{\lambda-\omega} - \frac{\lambda}{1+\lambda^2}\right] \mathrm{d}\Omega(\lambda), \tag{16}$$

where ω is in the UHP, $\mathbb{C}$ is Hermitian, $\mathbb{D}$ is Hermitian positive semidefinite, and $\mathrm{d}\Omega(\lambda)$ is a matrix-valued measure satisfying certain integrability conditions. (An analogous representation in the Laplace domain was recognized by Youla [4]; Beltrami connected this work to earlier results by Herglotz and Cauer [5, 19].) The values of $\mathbb{C}$ and $\mathbb{D}$ are specified by $\mathbb{T}$: $\mathbb{C} = \mathrm{Re}\,[i\mathbb{T}(i)]$ and $\mathbb{D} = \lim_{y\to\infty}[\mathbb{T}(iy)]$.

B. Alternative derivation of the integral representation of the main text

We saw in the previous section the general Herglotz representation

$$\omega\mathbb{T}(\omega) = \mathbb{C} + \mathbb{D}\omega + \int_{-\infty}^{\infty} \left[\frac{1}{\lambda-\omega} - \frac{\lambda}{1+\lambda^2}\right] \mathrm{d}\Omega(\lambda), \tag{17}$$

where $\mathbb{C} = \mathrm{Re}\,[i\mathbb{T}(i)]$ and $\mathbb{D} = \lim_{y\to\infty}[\mathbb{T}(iy)]$. The $\mathbb{T}$ matrix decays as $1/\omega^2$, which enables significant simplification of the representation. From Remark 2.8.3 and Theorem 2.4.2 of Ref. [18], the growth condition

$$\int_0^{\infty} \mathbf{x}^{\dagger}\mathbb{T}(iw)\mathbf{x}\,\mathrm{d}w \le \infty, \tag{18}$$

implies a simplified representation. This growth condition is satisfied by the $\mathbb{T}(\omega)$ matrix thanks to its quadratic decay at high frequencies. Then, the representation is [18]

$$\omega\mathbb{T}(\omega) = \int_{-\infty}^{\infty} \frac{\mathrm{d}\Omega(\lambda)}{\lambda-\omega}. \tag{19}$$

We can use the symmetry condition on $\omega\mathbb{T}(\omega)$ (the third condition above Eq. (16)) to identify conditions on the matrix-valued measure $\mathrm{d}\Omega$. Note that

$$\omega^*\mathbb{T}(\omega^*) = \int_{-\infty}^{\infty} \frac{\mathrm{d}\Omega^*(\lambda)}{\lambda-\omega^*}, \tag{20}$$

and

$$-\left[\omega\mathbb{T}(\omega)\right]_{-\omega^*} = -\int_{-\infty}^{\infty} \frac{\mathrm{d}\Omega(\lambda)}{\lambda+\omega^*} = -\int_{-\infty}^{\infty} \frac{\mathrm{d}\Omega(-\lambda)}{\lambda-\omega^*}. \tag{21}$$

For Eqs. (20,21) to be equal at all frequencies, then $\mathrm{d}\Omega$ must satisfy

$$\mathrm{d}\Omega(-\lambda) = -\mathrm{d}\Omega^*(\lambda). \tag{22}$$

We can use this condition to simplify the integral relation to positive frequencies only:

$$\begin{aligned}\omega\mathbb{T}(\omega) &= \int_0^{\infty} \frac{\mathrm{d}\Omega(\lambda)}{\lambda-\omega} + \int_{-\infty}^{0} \frac{\mathrm{d}\Omega(\lambda)}{\lambda-\omega} \\ &= \int_0^{\infty} \frac{\mathrm{d}\Omega(\lambda)}{\lambda-\omega} + \int_0^{\infty} \frac{\mathrm{d}\Omega(-\lambda)}{\lambda+\omega} \\ &= \int_0^{\infty} \left[\frac{\mathrm{d}\Omega(\lambda)}{\lambda-\omega} - \frac{\mathrm{d}\Omega^*(\lambda)}{\lambda+\omega}\right].\end{aligned} \tag{23}$$

As a matrix-valued measure, $\mathrm{d}\Omega$ is Hermitian. We can define its reciprocal and nonreciprocal parts as $\mathrm{d}\mathbb{X}$ and $\mathrm{d}\mathbb{Y}$, respectively, both of which will also be Hermitian. Then we have $\mathrm{d}\Omega^*(\lambda) = \mathrm{d}\mathbb{X}^*(\lambda) + \mathrm{d}\mathbb{Y}^*(\lambda) = \mathrm{d}\mathbb{X}^{\dagger}(\lambda) - \mathrm{d}\mathbb{Y}^{\dagger}(\lambda) = \mathrm{d}\mathbb{X}(\lambda) - \mathrm{d}\mathbb{Y}(\lambda)$, and the integral relation becomes

$$\begin{aligned}\omega\mathbb{T}(\omega) &= \int_0^{\infty} \left[\frac{\mathrm{d}\mathbb{X}(\lambda)+\mathrm{d}\mathbb{Y}(\lambda)}{\lambda-\omega} - \frac{\mathrm{d}\mathbb{X}(\lambda)-\mathrm{d}\mathbb{Y}(\lambda)}{\lambda+\omega}\right] \\ &= \int_0^{\infty} \left[\frac{2\omega}{\lambda^2-\omega^2}\mathrm{d}\mathbb{X}(\lambda) + \frac{2\lambda}{\lambda^2-\omega^2}\mathrm{d}\mathbb{Y}(\lambda)\right].\end{aligned} \tag{24}$$

We can divide both sides by ω to isolate the $\mathbb{T}(\omega)$ matrix on the left-hand side, and subsume the factors of 2 on the right-hand side into the measures. Then we have

$$\mathbb{T}(\omega) = \int_0^\infty \left[\frac{1}{\lambda^2 - \omega^2} \mathrm{d}\mathbb{X}(\lambda) + \frac{\lambda}{\omega(\lambda^2 - \omega^2)} \mathrm{d}\mathbb{Y}(\lambda)\right]. \tag{25}$$

Remember that this expression is for ω in the upper-half plane. We can take the limit as ω approaches the real line, but we must do so carefully: in any expression of the form $1/(\lambda - \omega)$, we cannot discard the imaginary part of the frequency, even as it goes to zero, as the imaginary part of the entire expression approaches that of a delta function (in a distributional sense). By contrast, in terms of the form $1/(\lambda + \omega)$ (for $\lambda \geq 0$) or $1/\omega$, the imaginary part can be dropped in the limit that it goes to zero. If we define $\gamma = 2 \operatorname{Im} \omega$ and $\omega = \operatorname{Re} \omega$ (overloading notation), then we can write:

$$\mathbb{T}(\omega) = \lim_{\gamma \to 0} \int_0^\infty \frac{1}{\lambda^2 - \omega^2 - i\gamma\omega} \left[\mathrm{d}\mathbb{X}(\lambda) + \frac{\lambda}{\omega} \mathrm{d}\mathbb{Y}(\lambda)\right], \tag{26}$$

which is equivalent to Eq. (3) of the main text, with the replacements $\lambda \to \omega_i$, $\mathrm{d}\mathbb{X}(\lambda) \to \mathbb{X}(\omega_i)\mathrm{d}\omega_i$, and $\mathrm{d}\mathbb{Y}(\lambda) \to \mathbb{Y}(\omega_i)\mathrm{d}\omega_i$.

Supplementary Note 4. PHYSICAL OSCILLATORS VS. MATHEMATICAL OSCILLATORS

In the main text we contrasted our $\mathbb{T}$-matrix "mathematical-oscillator" representation with the well-known "physical-oscillator" decompositions in use today. Here we detail the similarities and differences in these approaches. At the highest level, physical-oscillator approaches are meant to be efficient for simulation and modeling: for a *given* structure, can one identify a small number of parameters (e.g. normal- or quasinormal-mode coefficients, etc.) that accurately model the complete response of the system? Typically these models are highly nonlinear in the unknown parameters, but there are standard computational methods for finding them. Yet for problems of design, these representations are difficult or impossible to work with: there is not a single given structure, anymore, but instead a large class of structures. It is not known *a priori* how many resonances or modes may contribute; to be safe, very large numbers must be used. So one is left with large, highly nonlinear models to optimize over, without any beneficial mathematical structure. The "mathematical-oscillator" theory developed in the main text is well-suited to this scenario. The use of lossless (and hence narrow-linewidth) oscillators naturally also leads to a large numbers of parameters (matrix-valued oscillator coefficients), but these parameters have ideal properties from an optimization perspective: they are positive-definite, constrained in sum, and linear in the only degrees of freedom. In fact, this decomposition is quite *ill-suited* for modeling: one needs to invert a large, dense matrix at every frequency to get the corresponding coefficients, which is computationally prohibitive except for small structures. But for *design*, one never needs to do any matrix inversion, and the mathematical structure of the decomposition is far superior for optimization. Below, we provide the mathematical expressions supporting these qualitative assertions.

In electromagnetic scattering simulations, there are two primary classes of "physical-oscillator" approaches: coupled-mode theory (CMT) [20–24], and quasinormal-mode (QNM) theories [25–31]. It can be shown that the former can be derived from the latter in the limit of isolated, high-Q resonances with negligible non-resonant scattering contributions [32]. We will describe both of these constructions. We start with coupled-mode theory. In coupled-mode theory, there is a basis of resonant modes described by a matrix Ω, whose diagonal terms are *complex-valued* resonance frequencies of the modes, and whose off-diagonal terms describe coupling rates between each pair of modes. Each resonance has "'overlap coeffients" with each outgoing-wave channel, the matrix containing these elements is often referred to as K (or D, which is identical to K in reciprocal systems). Finally, there is typically a non-trivial background scattering matrix S_{bg} that contains non-resonant contributions to the scattering process. In full, in any general (reciprocal) coupled-mode theory, the scattering matrix is given by the expression [20, 32]

$$S = S_{\text{bg}} - iK \left(\Omega - \omega\right)^{-1} K^T, \tag{27}$$

subject to reciprocity and unitarity conditions given by

$$K^\dagger K = 2 \operatorname{Im} \Omega, \tag{28}$$

$$S_{\text{bg}} K^* = -K. \tag{29}$$

One can immediately see that a CMT model constructed from these three equations will be impossible to optimize over. The degrees of freedom are the matrices Ω, S_{bg}, and K, none of which are Hermitian (let alone positive definite).

Moreover, one cannot even presuppose any finite, constrained size of the matrices, as there is no sum rule constraining any norm of the entries.

One route towards using CMT models for understanding limits is to remove much of the complexity from Eqs. (27)–(29). If one assumes background scattering cannot occur (although note that even in Mie resonators it plays a quite important role [32]), then $S_{\text{bg}} = I$ and $K^* = -K$, such that one can rewrite the scattering matrix relation as

$$S = \mathbb{I} + iK\left(\Omega - \omega\right)^{-1} K^{\dagger}, \tag{30}$$

a form of the S-matrix that also arises in nuclear scattering theory [33, 34]. Next, one can assume that none of the modes are coupled, such that Ω is a diagonal matrix. (This condition will typically conflict with the requirement that $K^{\dagger}K = 2\,\mathrm{Im}\,\Omega$, but we ignore that for simplicity.) Finally, special quantities such as absorption (given by $\mathbb{I} - S^{\dagger}S$ [35]) can be written as:

$$A = \mathbb{I} - S^{\dagger}S = -4K\left(\Omega - \omega\right)^{-\dagger}\left[\mathrm{Im}\,\Omega\right]\left(\Omega - \omega\right)^{-1} K^{\dagger}, \tag{31}$$

after repeated use of $K^{\dagger}K = 2\,\mathrm{Im}\,\Omega$ and the matrix identity $\mathrm{Im}\left[X^{\dagger}YX\right] = X^{\dagger}\left[\mathrm{Im}\,Y\right]X$. From Eq. (31), one can integrate over all frequencies to simplify the interior matrix product involving frequencies (which is a diagonal matrix with Lorentzians along the diagonal) to a constant. Finally, one is left with an expression involving only the loss rates of each resonator and the number of resonances [36–38]. But what are these values? How large can they be? One is always left with more free unconstrained parameters. And the extreme limits of these models, where one wants to operate for fundamental limits, are precisely where the assumptions mentioned above (high-Q resonances, uncoupled resonances, frequency-independent K matrix, isolated resonances, no background processes, etc.) break down. By contrast, the utility of CMT for *modeling* complex electromagnetic structures has been a theoretical bounty for twenty years [20, 22–24, 39–44], and CMT is well-deserving of its popularity for such scenarios.

To move beyond the assumptions of CMT, expansions via quasinormal modes (QNMs) have become more popular in recent years. There are various expansion techniques, many of which can be shown to be equivalent [26]. We will assume a Maxwell equation of the form

$$(M - \omega^2\varepsilon)e = i\omega j, \tag{32}$$

where we assume a sufficiently high-resolution discretization of Maxwell's equations, for matrix M and diagonal matrix ε, unknown electric-field vector e, and free-current source vector j. The boundary conditions or PMLs are assumed to be encoded in the matrix M, as well as the curl–curl operator. The pair of matrices M and ε form generalized eigenproblem pairs according to $MU = \varepsilon U\Lambda$, where U are the eigenfields and Λ the squared eigenfrequencies. We can assume reciprocity, in which case $U^{-1} = U^T$. Inserting this eigendecomposition into our Maxwell equation and solving for the electric field yields

$$e = i\omega U\left(\Lambda - \omega^2\right)^{-1} U^T\varepsilon^{-1}j. \tag{33}$$

One can interpret Eq. (33) intuitively: $U^T\varepsilon^{-1}j$ is a decomposition of normalized free currents into modal fields, $\left(\Lambda - \omega^2\right)^{-1}$ is the resonant enhancement associated with real frequencies close to the resonant frequencies, and the final U on the left converts from the modal basis back to the original (real-space) basis. Superficially, Eq. (33) actually looks quite similar to the coupled-mode scattering-matrix equation of Eq. (27): a resonant amplification term inversely proportional to the differences between the complex-valued resonant frequencies and the real excitation frequencies, and frequency-independent matrices surrounding the resonant-amplification term. However, to connect to the "scattering channels" that bring energy into or out of such systems, one would need to pre- and post-multiply these matrices with Green's-function matrices that are highly frequency-dependent. Then, again, one is left with a complex set of degrees of freedom: the number of resonances (the size of Λ and number of columns of U), the locations of the resonant poles in the complex plane (the values of Λ), and the resonant field patterns (the values of the columns of U). There are almost no constraints on these degrees of freedom, except that the field patterns must be orthogonal in the unconjugated inner product, corresponding to $U^TU = \mathbb{I}$. There is no meaningful way to convert this representation to upper bounds or fundamental limits. Again, however, this representation is quite useful for modeling, with a number of exemplary successes over the past decade [25–31, 45].

To summarize: Eqs. (27,33) are the key "physical-oscillator" descriptions of classical scattering processes. At a glance, they share similarities with each other and with the $\mathbb{T}$-matrix representation of the main text: at a coarse level, each has a resonant-enhancement term and one or more matrices that can be described as a "coupling" matrix. With more granularity, however, there are crucial mathematical differences between the two expressions of Eqs. (27,33) with the $\mathbb{T}$ matrix expression. In the CMT and QNM approaches, all of the degrees of freedom (the resonant pole locations and the coupling matrices) are complex-valued quantities without any Hermiticity or positive-definiteness

qualities. Moreover, the number of resonances can never be constrained for the arbitrarily patterned nanophotonic systems of interest. By contrast, in the $\mathbb{T}$ matrix expansion, all of the "resonant poles" are approaching the real axis, there is an infinite set (one need not limit the number of "resonances"), the degrees of freedom (the scattering-oscillator strengths) are positive semidefinite, Hermitian matrices, and their sum is constrained, thanks to sum rules. Because their poles are *not* related to the normal- or quasinormal-mode eigenfrequencies, $\mathbb{T}$ matrix expansions are computationally expensive for a given structure. But in optimizations over *all possible* geometries, their mathematical structure is unique, and pays significant dividends.

Supplementary Note 5. LOW-FREQUENCY NEAR-FIELD SUM RULE FOR THE $\mathbb{T}$ MATRIX

In the NFRHT bound, we used the generalized polarizability for two half-spaces, $\alpha_{2\text{hs}} = 2$. To derive this result, we first consider a simple case of a single half-space interface parallel to the xy-plane, and the medium in $z < 0$ has permittivity $\varepsilon_1 = 1$ while the medium in $z > 0$ is our half-space scatterer that has permittivity ε_2. A general electrostatic source is located in the air side, and at $z = -d$ away from the interface. In electrostatics, away from the source and the interface, one can write $\boldsymbol{E} = -\nabla\psi$ where $\nabla^2\psi = 0$. At each z, ψ can be expressed with a 2D Fourier integral:

$$\psi(x,y,z) = \iint\limits_{-\infty}^{+\infty} \mathrm{d}k_x \mathrm{d}k_x \; \tilde{\psi}\left(k_x,k_y,k_z\right) e^{ik_x x + ik_y y}, \tag{34}$$

where $\tilde{\psi}$ is the 2D Fourier transform of ψ. Away from the source and the interface, one can solve the electrostatic Poisson's equation and obtain the expressions for the electric field:

$$\boldsymbol{E}(x,y,z) = \iint\limits_{-\infty}^{+\infty} \mathrm{d}k_x \mathrm{d}k_x \; \left(k_x,k_y,k_z\right) U\left(k_x,k_y\right) e^{ik_x x + ik_y y + ik_z z} \tag{35}$$

$$+ \iint\limits_{-\infty}^{+\infty} \mathrm{d}k_x \mathrm{d}k_x \; \left(k_x,k_y,-k_z\right) V\left(k_x,k_y\right) e^{ik_x x + ik_y y - ik_z z} \tag{36}$$

for $-d < z < 0$, and

$$\boldsymbol{E}(x,y,z) = \iint\limits_{-\infty}^{+\infty} \mathrm{d}k_x \mathrm{d}k_x \; \left(k_x,k_y,k_z\right) W\left(k_x,k_y\right) e^{ik_x x + ik_y y + ik_z z} \tag{37}$$

for $z > 0$, where U, V, and W are the plane-wave modal field amplitudes for the incoming, the reflected and the transmitted fields. Note that in electrostatics, not only k_x and k_y but also $k_z = i\sqrt{k_x^2 + k_y^2}$ are conserved across the interface.

To find $\mathbb{T}(\omega = 0)$, we need to find the relation between the polarization current $\boldsymbol{P} = \chi\boldsymbol{E}$ and the incident field $\boldsymbol{E}_{\text{inc}}$ in the region $z > 0$, which is essentially finding the Fresnel coefficients. What are the Fresnel coefficients in electrostatics? As pointed out in Ref. [46], Fresnel equations apply to statics, and for electrostatic sources:

$$r = \frac{V}{U} = \frac{\varepsilon_1 - \varepsilon_2}{\varepsilon_1 + \varepsilon_2} \tag{38}$$

$$t = \frac{W}{U} = \frac{2\varepsilon_1}{\varepsilon_1 + \varepsilon_2} \tag{39}$$

Importantly, note that the Fresnel coefficients are independent of k_x and k_y, and therefore after the inverse Fourier transform, we have $\boldsymbol{E} = t\boldsymbol{E}_{\text{inc}}$, and $\alpha = \chi t$. Similarly, the arguments expressing the fields with Fourier basis apply when we consider two parallel half-spaces separated by d_0, but the transmission coefficient needs to be substituted by that of two interfaces

$$t_{2\text{hs}} = \frac{t\left(1 + re^{2ik_z d}\right)}{1 - r^2 e^{2ik_z d_0}} \tag{40}$$

Using $\varepsilon_2 = 1 - \frac{\omega_p^2}{\omega^2}$ at $\omega \to 0$, one can obtain $t_{2\text{hs}}(\omega = 0) = \frac{2}{\varepsilon_2}$ and $\alpha_{2\text{hs}} = \chi t_{2\text{hs}} = 2$. Therefore the electrostatic $\mathbb{T}$ matrix for the bounding volume of two half-spaces is $\mathbb{T}(\omega = 0) = \alpha_{2\text{hs}}\mathbb{I}$ where $\alpha_{2\text{hs}} = 2$.

Supplementary Note 6. SCATTERING SIMULATION PARAMETERS FOR FIG. 1

In this section we provide the detailed simulation data and techniques for Fig. 1 of the main text. The elliptical cylinder has susceptibility $\chi = 4$, width $D_x = 2.4a$, and height $D_y = 1.6a$, where a is a scale factor for length normalization. To obtain accurate results using the simulation method we will introduce below, the sharp edge of the geometry need to be smoothed. In this example, the susceptibility distribution of the elliptical cylinder is expressed as

$$\chi(x,y) = \frac{\chi}{2}\left\{1 + \tanh\left[c_1\left(1 - \sqrt{\frac{x^2}{D_x^2} + \frac{y^2}{D_y^2}}\right)\right]\right\}, \tag{41}$$

where c_1 is inversely proportional to the width of the smoothed area along the circumference of the ellipse. For the full-wave simulation, we use our own direct solver utilizing a discrete dipole approximation (DDA) augmented by a Duan-Rokhlin quadrature [47, 48]. The simulation region is a square of side length $3.0a$. Discretization of the square region gives 192 grid points along both x and y direction. There are 501 frequency sampling points ranging from 0.02 to 1, in units of $2\pi c/a$. The $\mathbb{T}$ matrix is obtained from Eq. (5), which is $\mathbb{T} = -(\mathbb{G}_0 + \xi\mathbb{I})^{-1}$, where $\mathbb{G}_0$ is the vacuum Green's function matrix and $\xi = -\frac{1}{\chi}$, both defined on the volume of scatterer. We use 6th-order Duan-Rokhlin correction for accurate computation of G, guaranteeing accuracy of less than 0.01% error in the computed extinguished power of the structure, at all frequencies of interest.

For plotting the E_{scat} and $\mathbb{T}$ matrix elements, we select 3 random points inside the scatterer: $x_1 = (-0.79, -0.36)a$, $x_2 = (0.74, -0.12)a$, and $x_3 = (0.93, 0.31)a$, using the center of the ellipse as the origin. The incident field is a plane wave propagating along the y direction with the electric field polarized perpendicular to the plane.

Supplementary Note 7. OPTIMAL FREQUENCIES OF NFRHT FOR STATE-OF-THE-ART MATERIALS

In Ref. [49] we study optimal bulk Drude materials, deriving a "near-field Wien's law" and identifying peak spectral-HTC frequencies for such materials. The peak-HTC frequencies for the optimal bulk material (red asterisks in Fig. 2(d)) are $\omega_{\text{opt}} = 2.57\frac{k_B T}{\hbar}$ according to the near-field Wien's law, where $\hbar$ is reduced Planck's constant and k_B is Boltzmann constant.

Next, we studied optimal 2D heterostructures. We optimize over 2D materials with different in-plane conductivities, each parametrized by a combination of resonance frequencies and material loss rates. Furthermore, multiple different layers of 2D materials directly stacked together constitute 2D heterostructures and we focus on optimizing those with 1,2 and 3 different monolayers. We find optimal NFRHT efficiency is achieved with a single optimal layer of 2D material, and multiple stackings do not perform better. The spectral response of this structure as well as that of the optimal bulk Drude material are shown in Fig. 2(c). The exact data for optimal frequencies (red and blue asterisks in Fig. 2(d)) are listed below:

Temperature (K)	Optimal bulk Drude (eV)	Optimal 2D heterostructure (eV)
100	0.0222	0.0227
200	0.0444	0.0455
300	0.0665	0.0683
400	0.0887	0.0912
500	0.1109	0.1141
600	0.1331	0.1370
700	0.1552	0.1604
800	0.1774	0.1838
900	0.1996	0.2072
1000	0.2218	0.2291
1100	0.2440	0.2511
1200	0.2661	0.2730

[1] R. Carminati and J. C. Schotland, *Principles of Scattering and Transport of Light*. Cambridge University Press, July 2021.
[2] W. C. Chew, M. S. Tong, and B. Hu, "Integral equation methods for electromagnetic and elastic waves," *Synthesis Lectures on Computational Electromagnetics*, vol. 3, pp. 1–241, 2008.

[3] G. Raisbeck, "A definition of passive linear networks in terms of time and energy," *Journal of Applied Physics*, vol. 25, no. 12, pp. 1510–1514, 1954.

[4] D. Youla, L. Castriota, and H. Carlin, "Bounded real scattering matrices and the foundations of linear passive network theory," *IRE Transactions on Circuit Theory*, vol. 6, no. 1, pp. 102–124, 1959.

[5] E. Beltrami, "Linear dissipative systems, nonnegative definite distributional kernels, and the boundary values of bounded-real and positive-real matrices," *Journal of Mathematical Analysis and Applications*, vol. 19, no. 2, pp. 231–246, 1967.

[6] M. R. Wohlers, *Lumped and distributed passive networks: a generalized and advanced viewpoint*. Academic press, 1969.

[7] J. C. Willems, "Dissipative dynamical systems part ii: Linear systems with quadratic supply rates," *Archive for rational mechanics and analysis*, vol. 45, pp. 352–393, 1972.

[8] S. Boyd and L. O. Chua, "On the passivity criterion for lti n-ports," *International Journal of Circuit Theory and Applications*, vol. 10, no. 4, pp. 323–333, 1982.

[9] P. K. Mahanta, N. Yamin, and A. Zadehgol, "Passivity verification and enforcement—a review paper," *International Journal of Numerical Modelling: Electronic Networks, Devices and Fields*, vol. 31, no. 3, p. e2286, 2018.

[10] A. Srivastava, "Causality and passivity: From electromagnetism and network theory to metamaterials," *Mechanics of Materials*, vol. 154, p. 103710, 2021.

[11] R. Kalman, "Old and new directions of research in system theory," in *Perspectives in Mathematical System Theory, Control, and Signal Processing: A Festschrift in Honor of Yutaka Yamamoto on the Occasion of his 60th Birthday*, pp. 3–13, Springer, 2010.

[12] P. Triverio, S. Grivet-Talocia, M. S. Nakhla, F. G. Canavero, and R. Achar, "Stability, causality, and passivity in electrical interconnect models," *IEEE Transactions on Advanced Packaging*, vol. 30, no. 4, pp. 795–808, 2007.

[13] H. Khodavirdi and A. Srivastava, "The analytical structure of acoustic and elastic material properties," *Wave Motion*, vol. 108, p. 102837, 2022.

[14] T. Hartman, S. Jain, and S. Kundu, "Causality constraints in conformal field theory," *Journal of High Energy Physics*, vol. 2016, no. 5, pp. 1–44, 2016.

[15] H. M. Nussenzveig, *Causality and Dispersion Relations*. New York, NY: Academic Press, 1972.

[16] F. Gesztesy and E. Tsekanovskii, "On matrix-valued herglotz functions," *Math. Nachr.*, vol. 218, pp. 61–138, Oct. 2000.

[17] B. Fritzsche, B. Kirstein, and C. Mädler, "On matrix-valued Herglotz-Nevanlinna functions with an emphasis on particular subclasses," *Math. Nachr.*, vol. 285, pp. 1770–1790, Oct. 2012.

[18] A. Luger and M.-J. Y. Ou, "On applications of Herglotz-Nevanlinna functions in material sciences, I: classical theory and applications of sum rules," *arXiv:2202.13247*, Feb. 2022.

[19] W. Cauer, "The poisson integral of functions with positive real parts," *Bull. Am. Math. Soc.*, vol. 38, pp. 713–717, 1932.

[20] W. Suh, Z. Wang, and S. Fan, "Temporal coupled-mode theory and the presence of non-orthogonal modes in lossless multimode cavities," *IEEE J. Quantum Electron.*, vol. 40, pp. 1511–1518, Oct. 2004.

[21] H. A. Haus, *Waves and fields in optoelectronics*. Prentice-Hall, 1984.

[22] J. D. Joannopoulos, S. G. Johnson, J. N. Winn, and R. D. Meade, *Photonic crystals: molding the flow of light*. Princeton University Press, 2011.

[23] S. Fan, W. Suh, and J. D. Joannopoulos, "Temporal coupled-mode theory for the fano resonance in optical resonators," *J. Opt. Soc. Am.*, vol. 20, no. 3, p. 569, 2003.

[24] R. E. Hamam, A. Karalis, J. D. Joannopoulos, and M. Soljačić, "Coupled-mode theory for general free-space resonant scattering of waves," *Phys. Rev. A*, vol. 75, p. 053801, May 2007.

[25] E. S. C. Ching, P. T. Leung, A. Maassen van den Brink, W. M. Suen, S. S. Tong, and K. Young, "Quasinormal-mode expansion for waves in open systems," *Rev. Mod. Phys.*, vol. 70, pp. 1545–1554, Oct. 1998.

[26] P. Lalanne, W. Yan, K. Vynck, C. Sauvan, and J.-P. Hugonin, "Light interaction with photonic and plasmonic resonances," *Laser & Photonics Reviews*, vol. 12, no. 5, p. 1700113, 2018.

[27] C. Sauvan, J.-P. Hugonin, I. Maksymov, and P. Lalanne, "Theory of the spontaneous optical emission of nanosize photonic and plasmon resonators," *Physical Review Letters*, vol. 110, no. 23, p. 237401, 2013.

[28] E. Muljarov and W. Langbein, "Resonant-state expansion of dispersive open optical systems: Creating gold from sand," *Physical Review B*, vol. 93, no. 7, p. 075417, 2016.

[29] P. Lalanne, W. Yan, A. Gras, C. Sauvan, J.-P. Hugonin, M. Besbes, G. Demésy, M. Truong, B. Gralak, F. Zolla, *et al.*, "Quasinormal mode solvers for resonators with dispersive materials," *JOSA A*, vol. 36, no. 4, pp. 686–704, 2019.

[30] P. T. Kristensen, J. R. de Lasson, M. Heuck, N. Gregersen, and J. Mørk, "On the theory of coupled modes in optical cavity-waveguide structures," *Journal of Lightwave Technology*, vol. 35, no. 19, pp. 4247–4259, 2017.

[31] P. T. Kristensen, K. Herrmann, F. Intravaia, and K. Busch, "Modeling electromagnetic resonators using quasinormal modes," *Advances in Optics and Photonics*, vol. 12, no. 3, pp. 612–708, 2020.

[32] H. Zhang and O. D. Miller, "Quasinormal coupled mode theory," *arXiv preprint arXiv:2010.08650*, 2020.

[33] C. Mahaux and H. A. Weidenmüller, *Shell-model approach to nuclear reactions*. North-Holland Pub. Co., 1969.

[34] W. R. Sweeney, C. W. Hsu, and A. D. Stone, "Theory of reflectionless scattering modes," *Physical Review A*, vol. 102, no. 6, p. 063511, 2020.

[35] D. A. B. Miller, L. Zhu, and S. Fan, "Universal modal radiation laws for all thermal emitters," *Proc. Natl. Acad. Sci. U. S. A.*, vol. 114, no. 17, pp. 4336–4341, 2017.

[36] Z. Yu, A. Raman, and S. Fan, "Fundamental limit of nanophotonic light trapping in solar cells," *Proc. Natl. Acad. Sci. U. S. A.*, vol. 107, pp. 17491–17496, Oct. 2010.

[37] Z. Yu, A. Raman, and S. Fan, "Nanophotonic light-trapping theory for solar cells," *Appl. Phys. A: Mater. Sci. Process.*, vol. 105, pp. 329–339, Nov. 2011.

[38] Z. Yu, A. Raman, and S. Fan, "Thermodynamic upper bound on broadband light coupling with photonic structures," *Phys. Rev. Lett.*, vol. 109, p. 173901, Oct. 2012.
[39] L. Verslegers, Z. Yu, P. B. Catrysse, and S. Fan, "Temporal coupled-mode theory for resonant apertures," *J. Opt. Soc. Am. B*, vol. 27, p. 1947, Oct. 2010.
[40] L. Verslegers, Z. Yu, Z. Ruan, P. B. Catrysse, and S. Fan, "From electromagnetically induced transparency to superscattering with a single structure: A Coupled-Mode theory for doubly resonant structures," *Phys. Rev. Lett.*, vol. 108, p. 83902, Feb. 2012.
[41] C. W. Hsu, B. G. DeLacy, S. G. Johnson, J. D. Joannopoulos, and M. Soljačić, "Theoretical criteria for scattering dark states in nanostructured particles," *Nano Lett.*, vol. 14, pp. 2783–2788, May 2014.
[42] H. Zhou, B. Zhen, C. W. Hsu, O. D. Miller, S. G. Johnson, J. D. Joannopoulos, and M. Soljačić, "Perfect single-sided radiation and absorption without mirrors," *Optica*, vol. 3, pp. 1079–1086, Oct. 2016.
[43] C. W. Hsu, B. Zhen, A. D. Stone, J. D. Joannopoulos, and M. Soljačić, "Bound states in the continuum," *Nature Reviews Materials*, vol. 1, pp. 1–13, July 2016.
[44] S. A. Mann, D. L. Sounas, and A. Alù, "Nonreciprocal cavities and the time–bandwidth limit," *Optica*, vol. 6, pp. 104–110, Jan. 2019.
[45] M. Benzaouia, J. D. Joannopoulos, S. G. Johnson, and A. Karalis, "Quasi-normal mode theory of the scattering matrix, enforcing fundamental constraints for truncated expansions," *Phys. Rev. Research*, vol. 3, p. 033228, Sept. 2021.
[46] J. Skaar, "Fresnel's equations in statics and quasistatics," *European Journal of Physics*, vol. 40, p. 045201, Jun. 2019.
[47] M. A. Yurkin and A. G. Hoekstra, "The discrete-dipole-approximation code adda: Capabilities and known limitations," *Journal of Quantitative Spectroscopy and Radiative Transfer*, vol. 112, pp. 2234–2247, Sept. 2011.
[48] R. Duan and V. Rokhlin, "High-order quadratures for the solution of scattering problems in two dimensions," *J. Comput. Phys.*, vol. 228, p. 2152–2174, Apr. 2009.
[49] L. Zhang and O. D. Miller, "Optimal materials for maximum large-area near-field radiative heat transfer," *ACS Photonics*, vol. 7, no. 11, pp. 3116–3129, 2020.
